# Clinical characterization and genomic landscape of gynecological cancers among patients attending a Chinese hospital

**DOI:** 10.3389/fonc.2023.1143876

**Published:** 2023-03-30

**Authors:** Cen Jiang, Yiyi Lu, Hua Liu, Gang Cai, Zhao Peng, Weiwei Feng, Lin Lin

**Affiliations:** ^1^ Department of Laboratory Medicine, Ruijin Hospital, Shanghai Jiaotong University School of Medicine, Shanghai, China; ^2^ Department of Obstetrics & Gynecology, Ruijin Hospital, Shanghai Jiaotong University School of Medicine, Shanghai, China; ^3^ Genecast Biotechnology Co., Ltd., Wuxi, China

**Keywords:** gynecological cancer, next-generation sequencing, TMB, BRCA1, BRCA2, FGFR3

## Abstract

**Background:**

Gynecological cancers are the most lethal malignancies among females, most of which are associated with gene mutations. Few studies have compared the differences in the genomic landscape among various types of gynecological cancers. In this study, we evaluated the diversity of mutations in different gynecological cancers.

**Methods:**

A total of 184 patients with gynecological cancer, including ovarian, cervical, fallopian tube, and endometrial cancer, were included. Next-generation sequencing was performed to detect the mutations and tumor mutational burden (TMB). Kyoto Encyclopedia of Genes and Genomes (KEGG) and Gene Ontology (GO) enrichment analyses were also conducted.

**Results:**

We found that 94.57% of patients had at least one mutation, among which single nucleotide variants, insertions and InDels were in the majority. *TP53, PIK3CA, PTEN, KRAS, BRCA1, BRCA2, ARID1A, KMT2C, FGFR2*, and *FGFR3* were the top 10 most frequently mutated genes. Patients with ovarian cancer tended to have higher frequencies of *BRCA1* and *BRCA2* mutations, and the frequency of germline *BRCA1* mutations (18/24, 75.00%) was higher than that of *BRCA2* (11/19, 57.89%). A new mutation hotspot in *BRCA2* (I770) was firstly discovered among Chinese patients with gynecological cancer. Patients with *TP53*, *PIK3CA*, *PTEN*, and *FGFR3* mutations had significantly higher TMB values than those with wild-type genes. A significant cross was discovered between the enriched KEGG pathways of gynecological and breast cancers. GO enrichment revealed that the mutated genes were crucial for the cell cycle, neuronal apoptosis, and DNA repair.

**Conclusion:**

Various gynecological cancer types share similarities and differences both in clinical characterization and genomic mutations. Taken together with the results of TMB and enriched pathways, this study provided useful information on the molecular mechanism underlying gynecological cancers and the development of targeted drugs and precision medicine.

## 1 Introduction

Ovarian (OC), cervical (CC), and endometrial cancer (EC) are the most common gynecological cancers in the female reproductive system (1, 2). OC is the most lethal gynecological malignancy in developed countries (3), with a 5-year survival rate of ~47% (4). Since ovaries are relatively small and located deep in the pelvic cavity, up to 59% of OCs are only detected at advanced stages, with a low survival rate (5). Epithelial ovarian carcinoma (EOC) accounts for the majority of OCs and can be divided into serous, endometrioid, clear cell, and mucinous carcinoma, amongst others. Serous carcinomas constitute about 75% of EOCs and are further divided into low-grade and high-grade serous carcinomas (LGSC and HGSC) depending on their histological differences (6). CC is the fourth most common cancer among females, affecting approximately 600,000 women annually (7). Although screening programs and human papillomavirus (HPV) vaccines have helped reduce its incidence (8), approximately 310,000 patients with CC die annually (9). CC tends to develop at a younger age (10, 11); however, older patients also often have dismal prognoses (12, 13). EC, which is second only to CC in terms of the incidence of reproductive system cancers, ranks seventh among the most prevalent malignancies among females (14–16). EC can be divided into two types: type I estrogen-dependent EC (EEC) and type II non-estrogen-dependent EC (NEEC) (17). The proportion of patients with EEC is higher, and they are often younger and present with hypertension, diabetes, obesity, and infertility (18). NEEC has higher rates of metastasis and recurrence, poorer prognoses, and is more common among older women (19, 20). Fallopian tube cancer (FTC), which originates in the salpingeal mucosa (21), exhibits clinical behaviors similar to that of OC (22). But mutated fallopian tube epithelial cells were reported to form malignant tumors with a shorter latency and higher penetrance than that of ovarian surface epithelium. Although FTC is a relatively rare gynecological cancer, its incidence increased 4.19-fold from 2001 to 2014 (23).

Cancers are genetic diseases. Gene mutations alter the structure or function of related and encoded proteins, resulting in excessive/persistent stimulation signals for cell growth and transformation. With the development of molecular biology, the use of genetic testing to determine mutations in related tumors has become a topic of interest. Targeted drugs for specific genes and mutations are effective ways to treat cancer. Approximately 10 to 15% of OC are reported to be hereditary, and patients with OC are carriers of germline mutations in *BRCA1* and *BRCA2* (24). *BRCA1* and *BRCA2* mutations increase the lifetime risk of peritoneal malignancies and FTC (25, 26). In 2014, olaparib, the first poly ADP-ribose polymerase (PARP) inhibitor, was approved for the treatment of *BRCA*-mutated OC (27). *PPP2R1A* and *TP53* mutations are dramatically higher in patients with advanced-stage EC (19). *PIK3CA, KMT2C*, and *KMT2D* are the most frequently mutated genes in CC (28).

Next-generation sequencing (NGS) is a high-throughput sequencing technology that plays a vital role in cancer research (29). NGS can identify genomic alterations occurring in any region of a target gene, detect one mutated copy among thousands of wild-type copies, and elucidate many types of mutational landscapes of tumors. NGS has become an important aspect of accurate tumor diagnosis and treatment and has a variety of uses, such as tumor-targeted therapy-related driver gene detection, analysis of drug resistance mechanisms, tumor metastasis and prognosis assessment, and molecular diagnostics. In this study, we investigated 184 patients with gynecological malignancies using NGS and created a genomic landscape to show the diversity among different gynecological cancers, providing useful information for future clinical treatment.

## 2 Materials and methods

### 2.1 Patients and sampling

A total of 184 patients diagnosed with gynecological cancers at Ruijin Hospital, Shanghai Jiaotong University School of Medicine between January 2020 and June 2022 were enrolled in this study. All included patients gave their informed consent. The study was reviewed and approved by the Ethical Committee of the Ruijin Hospital, Shanghai Jiaotong University School of Medicine. Tissue samples were collected during surgical procedures and were subjected to NGS alongside paired blood samples. Patient information was acquired from medical records. Pathology diagnosis including the tumor site, pathological type, tumor differentiation grade, as well as Federation of International of Gynecologists and Obstetricians (FIGO) grade, were reviewed by two expert pathologists from the pathology department.

### 2.2 DNA extraction

Imprint cytology was performed to evaluate tumor purity before DNA extraction. Briefly, freshly cut surfaces of tissue specimens were gently pressed to glass slides. Then the slides was stained with hematoxylin and eosin (HE) after fixing with 95% of ethyl alcohol for 5–6 s. If the percentage of tumor cells was higher than 15%, the specimen was considered qualified for subsequent extraction and sequencing. Genomic DNA was extracted from fresh tumor tissue using the TIANamp Genomic DNA Kit (TIANGEN, China). Genomic DNA from peripheral blood lymphocytes (PBL) was extracted using a TGuide S32 Magnetic Blood Genomic DNA Kit (TIANGEN, China). The concentration of DNA was measured using a Qubit dsDNA HS Assay Kit (Thermo Fisher, USA), whereas the DNA quality was assessed using an Agilent 2100 BioAnalyzer (Agilent, USA). All extractions and assays were conducted according to the manufacturers’ instructions supplied in the respective kits used in this study.

### 2.3 Library preparation and sequencing

Genomic DNA extracted from each tumor or PBL sample was sheared with Covaris LE220 to a length of 200 bp, and fragmented DNA was used to construct a library using the KAPA Hyper Preparation Kit (Kapa Biosystems, USA). Target regions were captured using the HyperCap Target Enrichment Kit (Roche, Switzerland). The customized panel used in the capture process includes 543 genes (30), which are tumor-related major genes, and spans around a 1.67 MB genomic region of the human genome (**Supplemental Table 1**; Genecast Biotechnology Co., Ltd., Beijing, China). Bioinformatic analyses of these 543 genes were carried out at a College of American Pathologists (CAP)-certified laboratory (Genecast Biotechnology). Hybridization and washing were conducted according to the manufacturer’s protocol. The captured library was sequenced on the instrument of Illumina Novaseq 6000, which produces paired-end reads with the length of each end as 150bp, according to the manufacturer’s protocol.

Clean sequenced reads were mapped to the human reference genome (hg19) using BWA (v0.7.17) (31). VarDict (version 1.5.1) was used to call single nucleotide variant (SNV) mutations (32), whereas compound heterozygous mutations were merged using FreeBayes (version 1.2.0) (33). After annotation using ANNOVAR (2015 Jun17) (34), somatic mutations were selected based on the following standards: (i) located in intergenic/intronic regions; (ii) synonymous SNVs; (iii) allele frequency ≥ 0.002 in Exome Aggregation Consortum (ExAC) and genome aggregation database (gnomAD) (35, 36); (iv) allele frequency <0.05 in the tumor sample/allele frequency <0.01 in the plasma sample; (v) strand bias mutations in the reads; (vi) support reads <5; (vii) depth <30.

### 2.4 Tumor mutational burden calculation

Primarily, dynamic nonsynonymous mutations in the coding regions were selected for the following analysis of TMB, while driver gene mutations and germline alterations in the Single Nucleotide Polymorphism database (dbSNP) were removed. We filtered SNV mutations in all samples according to the following rules: (i) not splicing or exonic; (ii) depth <100 X/allele frequency <0.05; (iii) allele frequency ≥ 0.002 in the ExAC and gnomAD; and (iv) strand bias mutations in the reads. After quantification of the number of somatic nonsynonymous SNVs, the value was extrapolated to the whole exome using a validated algorithm (37). TMB, measured in mutations per Mb, was then calculated after obtaining absolute mutation counts against the mutation spots of the normal samples using the following formula:


$$\text{TMB}=\frac{\text{Absolute mutation counts}\times 1000000}{\text{Panel exonic base number}}$$

### 2.5 Gene ontology and Kyoto encyclopedia of genes and genomes pathway enrichment analyses

GO functional enrichment and KEGG pathway analyses were performed using DAVID tools (https://david.ncifcrf.gov/). For GO analysis, contigs were categorized, and their molecular functions, cellular components, and biological processes were statistically analyzed.

### 2.6 Protein interaction

Search Tool for the Retrieval of Interacting Genes/Proteins (STRING, version 11.0, https://string‐db.org) was used to analyze functional interactions with a confidence of 0.7. “Ovarian cancer,” “cervical cancer,” “fallopian tube cancer,” and “endometrial cancer” were used as keywords in Chilibot (http://www.chilibot.net/) to analyze the interaction between genes and different gynecological cancers, excluding abstract co-occurrence relationships.

### 2.7 Statistical analysis

Descriptive statistics were presented as the median (interquartile range; IQR) for continuous variables, and as numbers (percentages) for categorical data. All data were analyzed using SPSS version 26.0 (SPSS Inc., Chicago, IL, USA). Mann–Whitney and Kruskal–Wallis nonparametric tests were conducted for comparisons between groups. Pearson’s Chi-square test was used for categorical variables. The Benjamini–Hochberg procedure was conducted to derive significance for enrichment tests. Spearman’s correlation coefficients with a two-tailed *p* value were determined for correlation analyses; *p* < 0.05 indicated significance. Data are visualized in graphs produced using GraphPad Prism version 8.0 and R software version 4.0.5.

## 3 Results

### 3.1 The clinical features of the analyzed cohort

Among the 184 patients, 140 had OC, 12 had CC, 8 had FTC, and 24 had EC. Clinicopathological characteristics are presented in **Table 1**. The median age at diagnosis was 60 (50–67) years old.

**Table 1:** Clinical characterization of the population in this study.

	OC	CC	FTC	EC	*p* value
	(n = 140)	(n = 12)	(n = 8)	(n = 24)	
Age at diagnosis					*p*=0.008
Median	61 (51-67)	48 (39-60)	67 (61-70)	57 (46-63)	
≥ 55 years	91 (65.00%)	3 (25.00%)	7 (87.50%)	14 (58.33%)	
Menopausal status					*p*=0.206
Pre-menopausal	35 (25.00%)	5 (41.67%)	0 (0.00%)	7 (29.17%)	
Post-menopausal	105 (75.00%)	7 (58.33%)	8 (100.00%)	17 (70.83%)	
Tumor size					*p=*0.01
Median	5.0 (2.5-8.5)	3.1 (1.6-4.8)	3.4 (1.5-4.4)	4.0 (2.0-5.9)	
≥5 cm	75 (53.57%)	3 (25.00%)	1 (12.50%)	8 (33.33%)	
Metastasis					*p*=0.027
Node	23 (16.43%)	1 (8.33%)	0 (0.00%)	4 (16.67%)	
Organ	9 (6.43%)	2 (16.67%)	0 (0.00%)	1 (4.17%)	
Both	73 (51.14%)	4 (33.33%)	8 (100.00%)	7 (29.17%)	
None	35 (25.00%)	5 (41.67%)	0 (0.00%)	12 (50.00%)	
FIGO stage					*p=*0.000
I-II	39 (27.86%)	9 (75.00%)	3 (37.50%)	16 (66.67%)	
III-IV	101 (71.14%)	3 (25.00%)	5 (62.50%)	8 (33.33%)	
Personal history					
Breast cancer	8 (5.71%)	0 (0.00%)	0 (0.00%)	0 (0.00%)	
Thyroid cancer	2 (1.43%)	0 (0.00%)	0 (0.00%)	0 (0.00%)	
Hematologic tumor	2 (1.43%)	0 (0.00%)	0 (0.00%)	0 (0.00%)	
Renal cancer	1 (0.71%)	0 (0.00%)	0 (0.00%)	0 (0.00%)	
Liver cancer	1 (0.71%)	0 (0.00%)	0 (0.00%)	0 (0.00%)	
Colon cancer	1 (0.71%)	0 (0.00%)	0 (0.00%)	0 (0.00%)	
Family history					
Thyroid cancer	1 (0.71%)	0 (0.00%)	0 (0.00%)	0 (0.00%)	
Lung cancer	1 (0.71%)	0 (0.00%)	0 (0.00%)	0 (0.00%)	

OC, ovarian cancer; CC, cervical cancer; EC, endometrial cancer; FTC, fallopian tube cancer; FIGO, Federation of International of Gynecologists and Obstetricians.

Patients with CC were younger at diagnosis, especially compared with those with OC and FTC. No significant differences were observed in menopausal status. Patients with OC had larger tumors, found at more advanced stages, while patients with CC and EC were diagnosed at earlier stages (*p* < 0.05). Metastasis occurred at both at node and organs in all patients with FTC. Patients with OC tended to have personal/family histories of cancer, especially breast cancer. Further analysis was performed among patients with OC according to their pathological types (**Supplementary Table 2**).

### 3.2 Gynecological cancers exhibit various genomic landscapes

**Figure 1:**
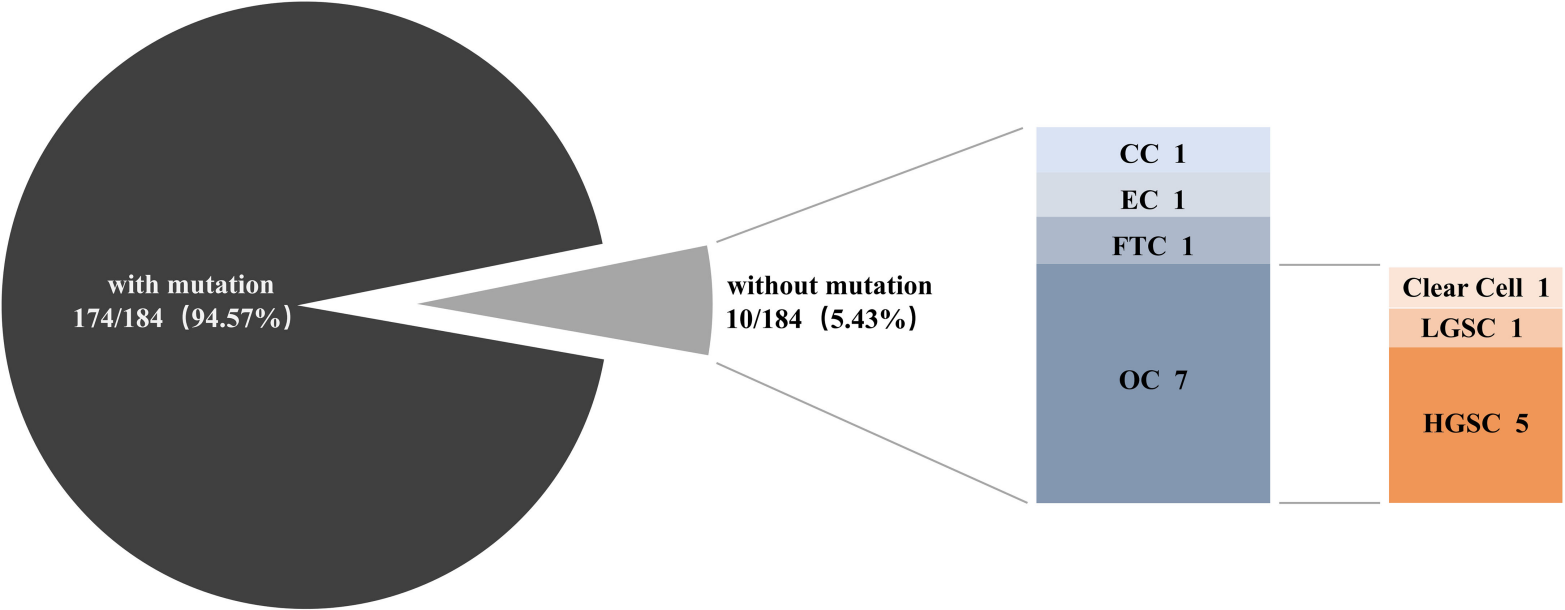
The proportion of patients with and without mutations. Next-generation sequencing was performed among 184 gynecological cancer patients to detect genomic alterations. OC, ovarian cancer; CC, cervical cancer; EC, endometrial cancer; FTC, fallopian tube cancer; LGSC, low-grade serous cancer; HGSC, high-grade serous cancer.

**Figure 2:**
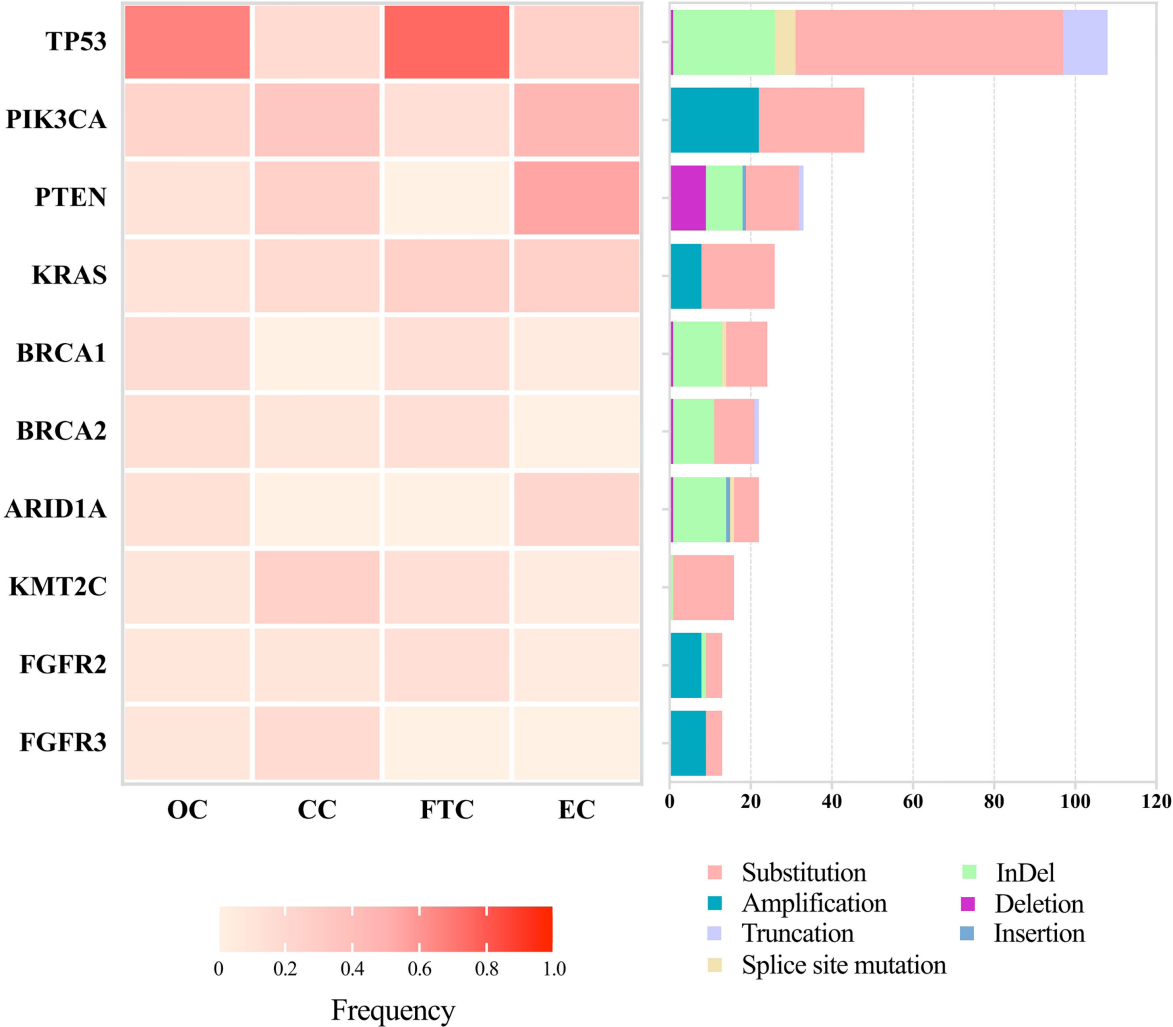
Landscapes of the top 10 most frequently mutated genes among 184 patients with gynecological cancer. Next-generation sequencing was performed to detect mutations. Frequencies of mutated genes are listed on the left, and mutation types are shown on the right, with annotation bars at the bottom. OC, ovarian cancer; CC, cervical cancer; EC, endometrial cancer; FTC, fallopian tube cancer.

Patient DNA from tumor tissues and matched peripheral blood were used for NGS. We detected 529 SNVs, 132 insertions and InDels, 36 truncations, 111 gene amplifications, 36 gene deletions, and 17 splice site mutations. Of all our patients, 94.57% (174/184) had at least one mutation (**Figure 1**). The top 10 most frequently altered genes in patients with gynecological cancer are presented in **Figure 2**. Patients with OC and FTC had higher frequencies of *TP53* mutations, while patients with EC showed more *PTEN* alterations. Changes in *KMT2C* and *FGFR3* were more frequent among patients with CC than in the other three types.

Furthermore, different mutation types were uncovered among different genes. *TP53* showed obvious alterations in SNVs and InDels. *PIK3CA* and *PTEN* revealed higher frequencies of copy number variations. *BRCA1* and *BRCA2* had similar patterns with slight differences, such as splice site mutations in *BRCA1* and insertions in *BRCA2*. R273 and V173 in *TP53*, H1047 and E542 in *PIK3CA*, R183 in *PPP2R1A*, and G12 in *KRAS* were hotspots of mutations among these patients (data not shown). The top 10 most frequently altered genes among patients with OC were the same as those in all 184 patients, but in an order with slight changes (**Supplementary Figure 1**).

### 3.3 Analysis of *BRCA1* and *BRCA2* mutations

**Figure 3:**
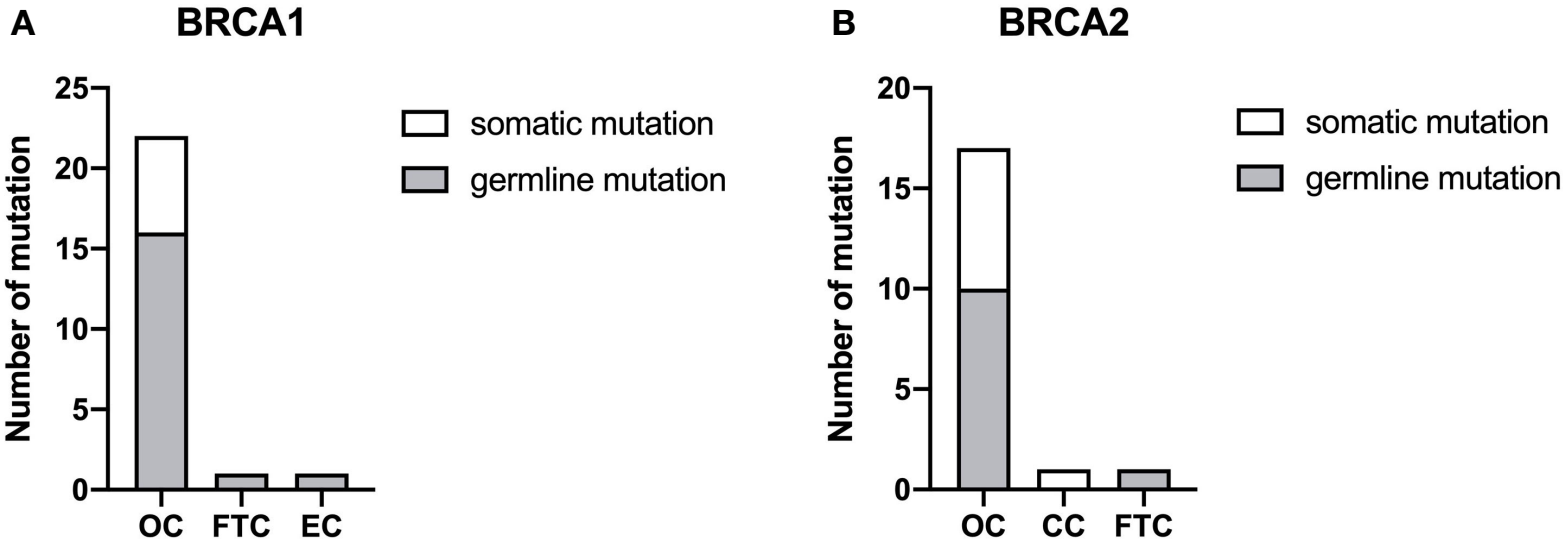
The proportion of *BRCA*1 **(A)** and *BRCA2* **(B)** mutations. Somatic and germline mutations were respectively detected by next-generation sequencing among the different types of gynecological cancer. OC, ovarian cancer; CC, cervical cancer; EC, endometrial cancer; FTC, fallopian tube cancer.

In total, 24 and 19 mutations were discovered in *BRCA1* and *BRCA2*, respectively, most of which were found in patients with OC (**Figure 3**). Two patients with OC (HGSC and endometrioid carcinoma, respectively) carried both *BRCA1* and *BRCA2* mutations simultaneously. No *BRCA1* or *BRCA2* mutations were found in patients with CC. The proportion of germline mutations was higher than somatic mutations in *BRCA1* and *BRCA2*. Moreover, the frequency of germline *BRCA1* mutations (18/24, 75.00%) was higher than that of *BRCA2* mutations (11/19, 57.89%). HGSC accounted for the majority of germline mutations in both *BRCA1* and *BRCA2* in patients with OC (**Supplementary Figure 2**). *BRCA2* mutation c.2307delT p.I770Ffs*2 was the hotspot firstly reported here among Chinese patients with gynecological cancer.

### 3.4 TMB analysis

**Figure 4:**
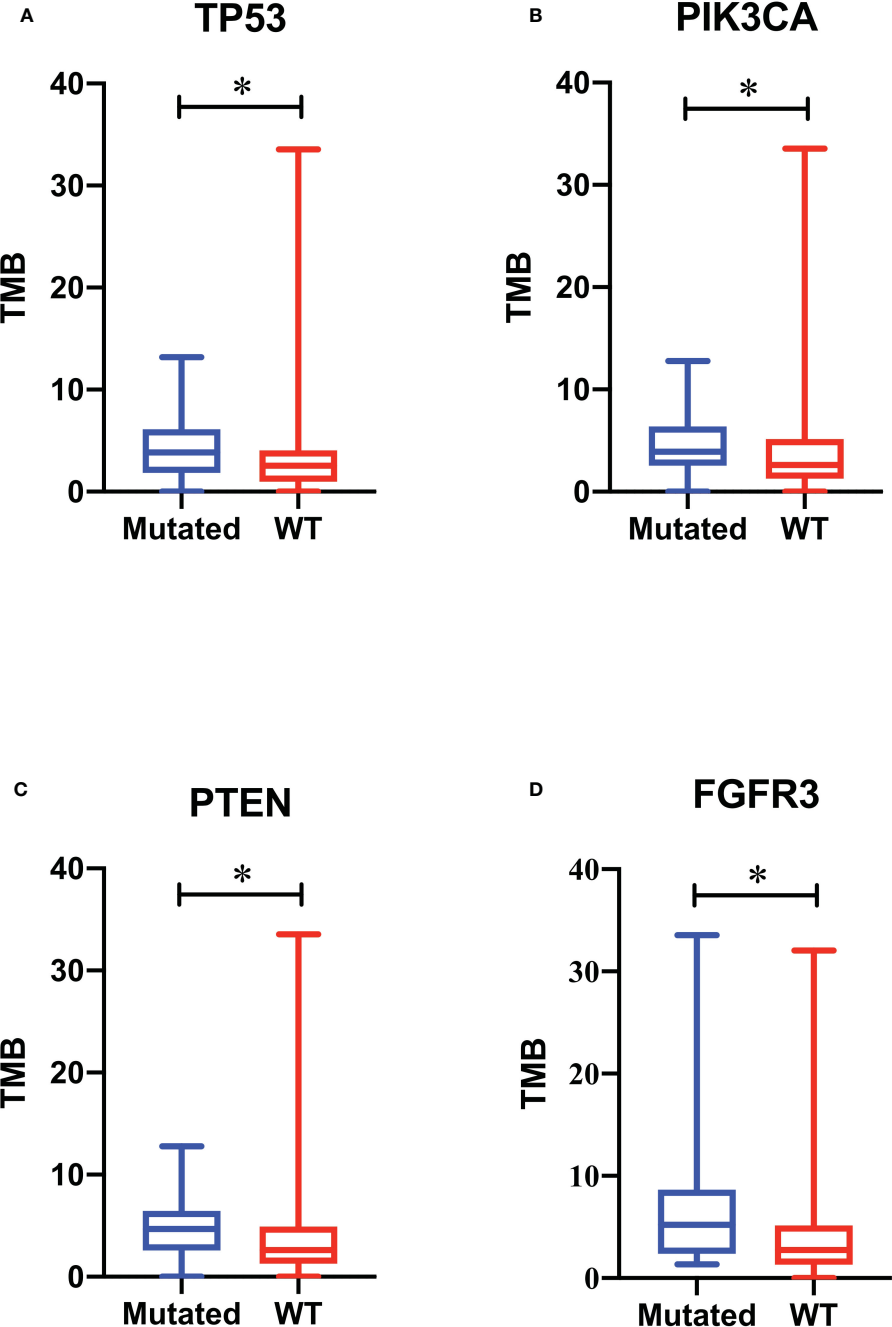
Association of gene mutations with tumor mutational burden (TMB). TMB values of patients with *TP53* **(A)**, PIK3CA **(B)**, *PTEN* **(C)**, and *FGFR3* **(D)** mutations are respectively compared with those of patients with wild-type genes. A box plot was used to show the minimum, maximum, median, and interquartile range of the TMB values. The blue box represents patients with mutations, and the red box represents patients with wild-type genes. * *p* < 0.05.

TMB range in this study spanned 0 to 192.35. To improve the accuracy, the data from one patient with endometrioid OC with statistical outliers (TMB = 192.35) was removed. The median TMB for all remaining patients was 2.94 (1.34–5.17). We ranked the TMB values from the lowest to highest and classified them into low, moderate, and high categories using quantiles ≤ 25%, 25–75%, and ≥75%, respectively. The ratio for TMB-low, TMB-moderate, and TMB-high was 32.79% (60/183), 54.64% (100/183), and 12.57% (23/183), respectively. No difference was observed between the median of the four gynecological cancer types (*p* = 0.200, **Table 2**); however, patients with EC tended to have a higher ratio of TMB-high values. Further analysis of TMB among patients with OC is shown in **Supplementary Table 3**. No correlation was observed between TMB and age, tumor size, menopausal status, metastasis, or FIGO stage (data not shown). Further, we analyzed the association between TMB and the top 10 most frequently changed genes in **Figure 2**. Compared with the wild-type, significant differences were discovered in the median of TMBs among patients with *TP53*, *PIK3CA*, *PTEN*, and *FGFR3* mutations (*p* < 0.05, **Figure 4**).

**Table 2:** Tumor mutational burden of the population in this study.

	OC	CC	FTC	EC	*p* value
	(n = 139)	(n = 12)	(n = 8)	(n = 24)	
Median	2.94 (1.30-5.17)	2.10 (0.00-6.02)	2.76 (1.91-4.19)	3.85 (2.56-7.44)	*p*=0.200
Low	50 (35.97%)	6 (50.00%)	2 (25.00%)	2 (8.33%)	
Moderate	74 (53.24%)	4 (33.33%)	6 (75.00%)	16 (66.67%)	
High	15 (10.79%)	2 (16.67%)	0 (0.00%)	6 (25.00%)	

OC, ovarian cancer; CC, cervical cancer; EC, endometrial cancer; FTC, fallopian tube cancer.

### 3.5 Enrichment analysis and protein interaction

**Figure 5:**
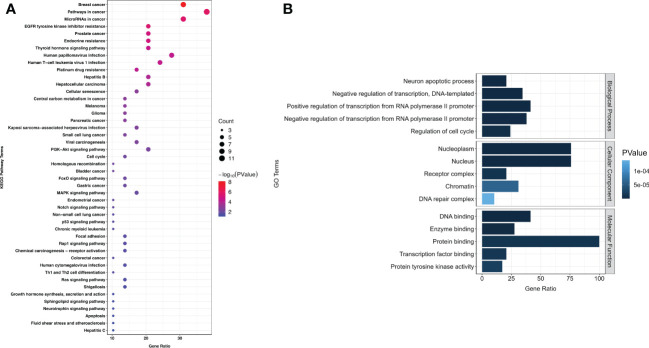
KEGG **(A)** and GO **(B)** enrichment among 184 patients with gynecological cancer. Next-generation sequencing was performed to detect mutations. Overlap of SNVs- and insertions and InDels-associated 29 genes was conducted for KEGG and GO enrichment. The size of each dot in KEGG enrichment indicates the number of genes included. The bigger the dot, the more genes are involved in the pathway. The top five GO enrichments are listed according to their *p* values.

**Figure 6:**
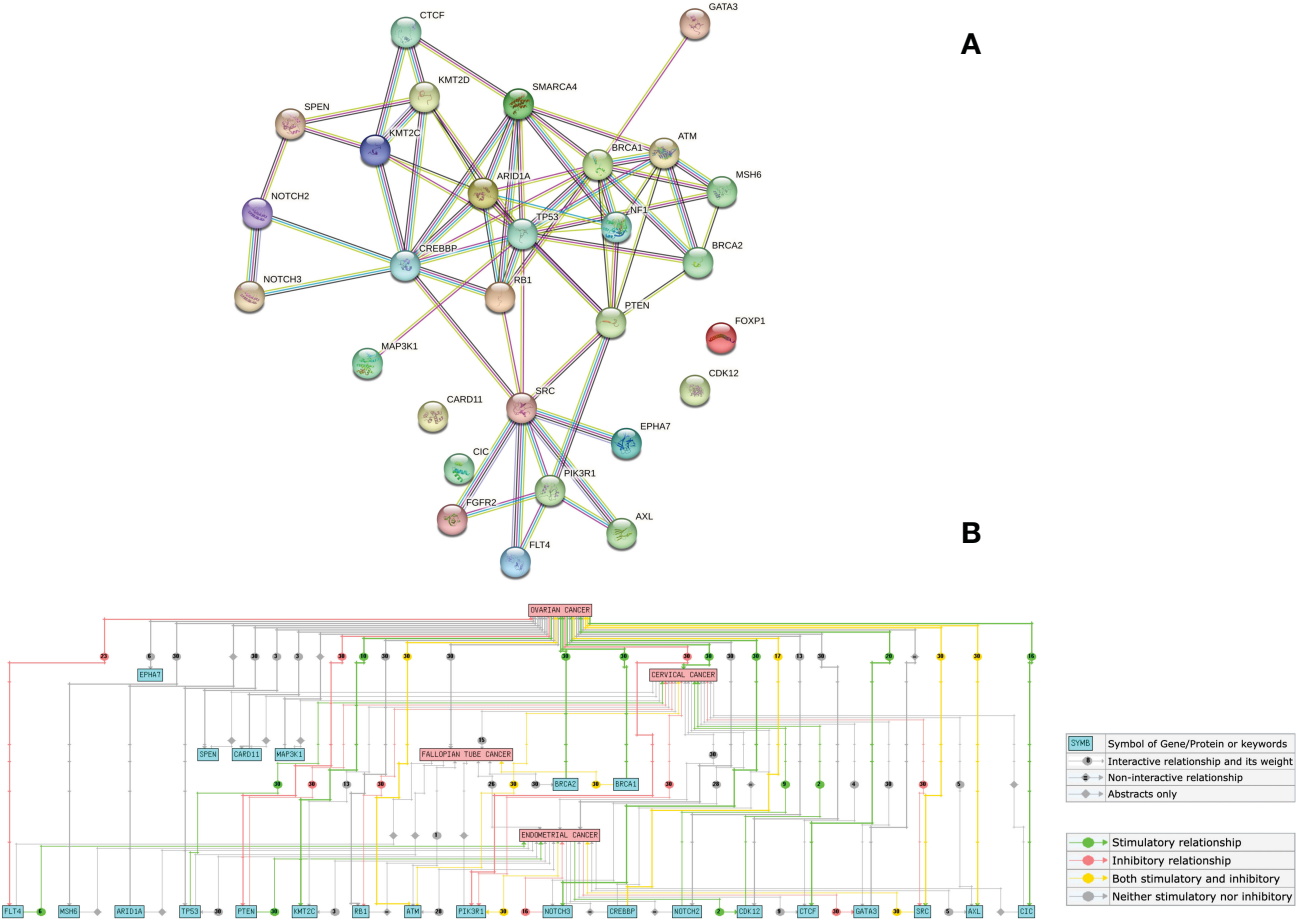
Interaction between mutated genes. Overlap analysis of SNVs- and insertions and InDels-associated 29 genes were performed on STRING **(A)** and Chilibot **(B)**. A confidence of 0.7 was used to analyze functional interactions on the STRING website. “Ovarian cancer”, “cervical cancer”, “fallopian tube cancer”, and “endometrial cancer” were used as keywords on Chilibot to analyze the interaction between genes and different gynecological cancers, excluding abstract co-occurrence relationships.

In this cohort, 529 SNVs and 132 insertions and InDels were detected, which accounted for the majority of mutations (661/861, 76.77%). Therefore, we performed an overlap analysis of the SNVs- and insertions and InDels-associated 29 genes. The KEGG and GO analyses of these genes are shown in **Figure 5**. A significant cross was discovered between the enriched pathways of gynecological and breast cancers (**Figure 5A**). Enrichment also revealed potential resistance to epidermal growth factor receptor (EGFR) tyrosine kinase inhibitors, endocrine, and platinum drugs. The top five enriched GO terms in biological processes, cellular components, and molecular functions are listed according to their *p* values (**Figure 5B**). Results showed that the mutated genes were crucial for neuronal apoptosis and DNA repair, as well as normal cell cycle. Protein interaction analysis identified *TP53* as a crucial protein in the network (**Figure 6A**). *SRC, RB1, CREBBP, ARID1A, SMARCA4, BRCA1*, and *ATM* also contributed significantly to the interaction net. Chilibot analysis showed that most of these mutated genes had stimulatory or inhibitory relationships with different gynecological cancers (**Figure 6B**).

## 4 Discussion

Gynecological cancers are among the most common malignancies with significant morbidity and mortality, primarily classified into five major types according to the organ affected (38, 39). In this retrospective study, we investigated 184 Chinese patients with gynecological cancer using NGS. Patients with OC, CC, EC, and FTC shared similarities but also varied both in clinical characterization and genomic landscape. It is worth noting that our research also has some limitations. Firstly, consistent with their clinical incidence, the numbers of patients with CC, EC, and FTC were limited in this study. Therefore, the relevant statistical results among patients with OC were more informative. Secondly, no overall survival data are provided at this time since the most recent patient enrolled was in June 2022. Therefore, this study may provide preliminary information for the clinical features and mutations of different types of gynecological tumors, and offer new ideas for future clinical treatment and targeted drug development.

It was reported that women with hereditary breast cancer have a 30–50% chance of developing OC (40). All the patients with a family and personal history of cancer in this study were diagnosed with OC, especially those with breast cancer. Our KEGG enrichment results also showed a significant cross between gynecological cancers and breast cancer (**Figure 5A**). Therefore, for persons with family history, especially with breast cancer history, it is crucial they undergo gynecological tumor gene screening as early as possible.

NGS technology gives us an opportunity to rapidly sequence multiple genes simultaneously and discover relevant mutations to guide treatment, which is beneficial in the field of precision or personalized medicine (41). *TP53*, the most frequently mutated gene in OC and FTC in our study (**Figure 2**), was reported in 1979 as the earliest gene to be associated with gynecological cancer (42). Consistently, an analysis from The Cancer Genome Atlas demonstrated that 96% HGSC was characterized by *TP53* mutation (43). *KMT2C* and *FGFR3* mutations had higher frequencies among the patients with CC in our study (**Figure 2**), of which FGFR3-TACC3 fusion was recently reported to be a potential molecular mechanism for inducing small cell cervical carcinoma (44). *PTEN* overexpression was suggested to promote morular differentiation in EC (45), but our results showed that *PTEN* deletion also played an important role (**Figure 2**). SNV was the most common mutation in our study, followed by insertions and InDels. A combination of these mutation-associated genes and the top 10 most frequent mutations will constitute the potential multi-gene panel to screen gynecological cancers.

*BRCA1* and *BRCA2* mutations are related to the DNA double-strand break repair process, which is also demonstrated in the GO enrichment result (**Figure 5B**). The process will not proceed normally when these two genes mutate, and the upstream codon will be converted to a stop codon and thus affect the protein formation (40). *BRCA* gene mutations are also indicators for PARP inhibitor (43, 46) and chemotherapy treatment (47). To the best of our knowledge, the mutation hotspot in *BRCA2* (I770) discovered in our study is the first reported among Chinese patients with gynecological cancer (48–50). Patients with *BRCA2* mutations have a better prognosis than those with *BRCA1* mutations (51).

Comprehensive understanding of factors associated with genomic instability is crucial for improving our knowledge of carcinogenesis. TMB is defined as the total number of somatic coding mutations, base substitutions, and insertion–deletion errors per million bases (52). Recently, researchers have identified the crucial role of TMB in response to immunotherapy and patient prognosis (53, 54). Higher TMB is associated with higher-grade, advanced clinical stage, and immunosuppressive phenotypes (55). According to our results, patients with EC (**Table 2**) and mucinous carcinoma (**Supplementary Table 3**) tended to have a higher ratio of TMB-high values. Zhu et al. also documented that the TMB of mucinous tumors in their study was higher than that of HGSC and LGSC (56). However, limited by the sample sizes in our study, more patient data in a larger cohort will be collected to verify this conclusion. A TMB value ≥75% level is usually defined as TMB-high (57), and there were 23 (12.57%) patients with TMB-high in this study. Pre-menopause was found to contribute significantly to higher TMB values among these 23 patients (*p* < 0.05, data not shown). Genomic alterations are also documented to be associated with TMB. In this study, besides the most frequently altered genes *TP53*, *PKI3CA*, and *PTEN*, patients with *FGFR3* mutations also tended to have higher TMB values than those with wild-type genes (**Figure 4**). Erdafitinib has been approved for patients with urothelial carcinomas with select *FGFR3* mutations (58). Therefore, *FGFR3* may also become a potential target for patients with gynecological cancers.

## 5 Conclusions

In summary, our study elucidated the distinct genomic landscapes of various types of gynecological cancers. Taken together with the results of TMB and enriched pathways, this study preliminarily sheds light on the molecular mechanisms of gynecological cancers, and the information gained may contribute to the development of targeted drugs and clinical treatment in precision medicine. Further large-scale and multi-center studies will be performed to validate our findings.

## Data availability statement

The original contributions presented in the study are included in the article/**Supplementary Material**. Further inquiries can be directed to the corresponding authors.

## Ethics statement

The studies involving human participants were reviewed and approved by Ethics Committee of the Ruijin Hospital, Shanghai Jiao Tong University School of Medicine. The patients/participants provided their written informed consent to participate in this study.

## Author contributions

CJ and LL contributed to study conception and design. HL and WF performed surgeries and enrollment of patients. CJ and YL conducted patient recruitment, data collection and sequencing. ZP and GC performed bioinformatics analysis. CJ and YL drafted the manuscript. WF and LL revised the manuscript. All authors contributed to the article and approved the submitted version.
