# Supplementary material for: Clinical characterization and genomic landscape of gynecological cancers among patients attending a Chinese hospital
Source: Front Oncol. 2023 Mar 30;13:1143876. doi: 10.3389/fonc.2023.1143876 (PMC10101327; doi:10.3389/fonc.2023.1143876)
Supplement: Supplementary file 1 [file Image_1.pdf]

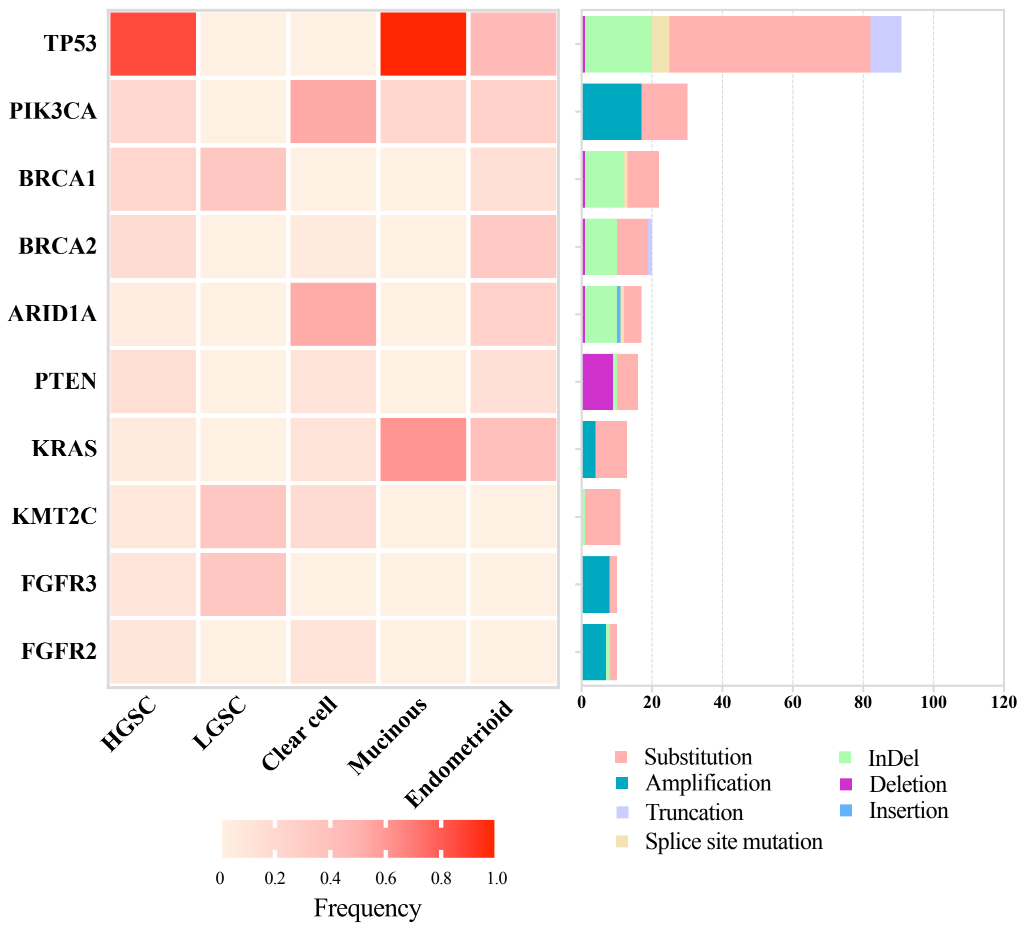

Supplementary Figure 1. Landscapes of the top 10 most frequently mutated genes among patients with ovarian cancers. Next-generation sequencing was performed to detect mutations. Frequency of mutated genes is listed in the left, and mutation types are showed in the right, with annotation bars at bottom. Results of nine patients whose pathological types don't belong to these five classifications are not shown. HGSC, high-grade serous carcinomas; LGSC, low-grade serous carcinomas.
