# Supplementary material for: Clinical characterization and genomic landscape of gynecological cancers among patients attending a Chinese hospital
Source: Front Oncol. 2023 Mar 30;13:1143876. doi: 10.3389/fonc.2023.1143876 (PMC10101327; doi:10.3389/fonc.2023.1143876)
Supplement: Supplementary file 2 [file Image_2.pdf]

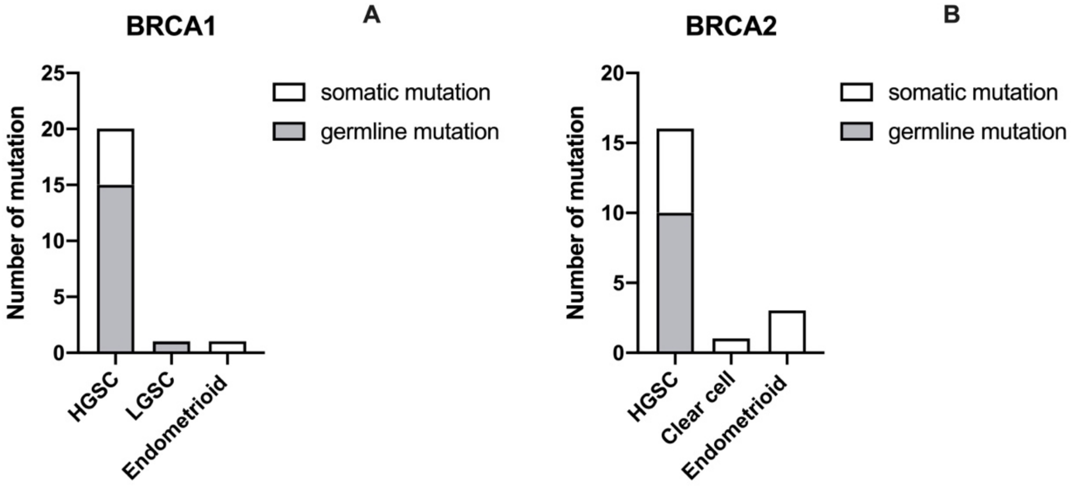

Supplementary Figure 2. The proportion of *BRCA1* (A) and *BRCA2* (B) mutation. Somatic and germline mutations were respectively detected by next-generation sequencing among patients with ovarian cancer according to their pathological types. HGSC, high-grade serous carcinomas; LGSC, low-grade serous carcinomas.
