# Supplementary material for: Clinical characterization and genomic landscape of gynecological cancers among patients attending a Chinese hospital
Source: Front Oncol. 2023 Mar 30;13:1143876. doi: 10.3389/fonc.2023.1143876 (PMC10101327; doi:10.3389/fonc.2023.1143876)
Supplement: Supplementary file 4 [file Table_2.pdf]

Supplementary Table 2 Clinical characterization of patients with ovarian cancer in this study [a]

	HGSC (n = 96)	LGSC (n = 3)	Clear cell (n = 19)	Mucinous (n=5)	Endometrioid (n = 8)	*p* value
**Age at diagnosis**						*p*=0.001
Median	64 (53-68)	48 (41-56)	55 (50-61)	47 (36-61)	49 (41-59)	
≥ 55 years	69 (71.88%)	1 (33.33%)	10 (52.63%)	2 (40.00%)	4 (50.00%)	
**Menopausal status**						*p*=0.003
Pre-menopausal	16 (16.67%)	2 (66.67%)	6 (31.58%)	3 (60.00%)	5 (62.50%)	
Post-menopausal	80 (83.33%)	1 (33.33%)	13 (68.42%)	2 (40.00%)	3 (37.50%)	
**Tumor size**						*p*=0.004
Median	4.0 (2.3-8.0)	6.5 (5.0-12.0)	11.0 (4.0-16.0)	7.0 (3.7-12)	8.0 (4.0-15)	
≥5 cm	47 (48.96%)	3 (100.00%)	13 (68.42%)	4 (80.00%)	5 (62.50%)	
**Metastasis**						*p*=0.000
Node	17 (17.71%)	1 (33.33%)	1 (5.26%)	1 (20.00%)	3 (37.50%)	
Organ	5 (5.21%)	0 (0.00%)	1 (5.26%)	0 (0.00%)	1 (12.50%)	
Both	65 (67.71%)	1 (33.33%)	1 (5.26%)	1 (20.00%)	1 (12.50%)	
None	9 (9.38%)	1 (33.33%)	16 (84.21%)	3 (60.00%)	3 (37.50%)	
**FIGO stage**						*p*=0.000
I-II	8 (8.33%)	1 (33.33%)	16 (84.21%)	3 (60.00%)	7 (87.50%)	
III-IV	88 (91.67%)	2 (66.67%)	3 (15.79%)	2 (40.00%)	1 (12.50%)	
**Personal history**						
Breast cancer	4 (4.17%)	1 (33.33%)	1 (5.26%)	0 (0.00%)	1 (12.50%)	
Thyroid cancer	2 (2.08%)	0 (0.00%)	0 (0.00%)	0 (0.00%)	0 (0.00%)	
Hematologic tumor	1 (1.04%)	0 (0.00%)	1 (5.26%)	0 (0.00%)	0 (0.00%)	
Renal cancer	1 (1.04%)	0 (0.00%)	0 (0.00%)	0 (0.00%)	0 (0.00%)	
Liver cancer	1 (1.04%)	0 (0.00%)	0 (0.00%)	0 (0.00%)	0 (0.00%)	
Colon cancer	1 (1.04%)	0 (0.00%)	0 (0.00%)	0 (0.00%)	0 (0.00%)	
**Family history**						
Thyroid cancer	1 (1.04%)	0 (0.00%)	0 (0.00%)	0 (0.00%)	0 (0.00%)	
Lung cancer	0 (0.00%)	0 (0.00%)	0 (0.00%)	0 (0.00%)	1 (12.50%)	

HGSC, high-grade serous carcinomas; LGSC, low-grade serous carcinomas; FIGO, Federation of International of Gynecologists and Obstetricians.

[a] Results of nine patients whose pathological types don’t belong to these five classifications are not shown.
