# Supplementary material for: Clinical characterization and genomic landscape of gynecological cancers among patients attending a Chinese hospital
Source: Front Oncol. 2023 Mar 30;13:1143876. doi: 10.3389/fonc.2023.1143876 (PMC10101327; doi:10.3389/fonc.2023.1143876)
Supplement: Supplementary file 5 [file Table_3.pdf]

Supplementary Table 3. Tumor mutational burden of patients with ovarian cancer in this study [a]

	HGSC n=96	LGSC n=3	Clear cell n=19	Mucinous n=5	Endometrioid n=7	*p* value
**Median**	3.26(1.36-5.81)	0.96(0.00-3.90)	3.64(1.41-4.84)	3.86(1.15-9.23)	1.65(0.98-2.61)	*p*=0.200
Low	32 (33.33%)	2 (66.67%)	5 (26.32%)	2 (40.00%)	4 (57.14%)	
Moderate	52 (54.17%)	1 (33.33%)	13 (68.42%)	2 (40.00%)	3 (42.86%)	
High	12 (12.50%)	0 (0.00%)	1 (5.26%)	1 (20.00%)	0 (0.00%)	

HGSC, high-grade serous carcinomas; LGSC, low-grade serous carcinomas.

[a] Results of nine patients whose pathological types don't belong to these five classifications are not shown.
